# Population pharmacokinetics of treosulfan and development of a limited sampling strategy in children prior to hematopoietic stem cell transplantation

**DOI:** 10.1007/s00228-017-2344-x

**Published:** 2017-10-03

**Authors:** Dorota Danielak, Jadwiga Twardosz, Anna Kasprzyk, Jacek Wachowiak, Krzysztof Kałwak, Franciszek Główka

**Affiliations:** 1 Department of Physical Pharmacy and Pharmacokinetics, Poznan University of Medical Sciences, Święcickiego 6 St, 60-781, Poznań, Poland; 2 Department of Pediatric Hematology, Oncology and Transplantology, Poznan University of Medical Sciences, Poznań, Poland; 3 Department of Pediatric Hematology, Oncology and Bone Marrow Transplantation, Wroclaw Medical University, Wrocław, Poland

**Keywords:** Hematopoietic stem cell transplantation, Area under curve, Infusions, intravenous, Population pharmacokinetics

## Abstract

**Purpose:**

There is an increasing interest in use of treosulfan (TREO), a structural analogue of busulfan, as an agent in conditioning regimens prior to hematopoietic stem cell transplantation (HSCT), both in pediatric and adult populations. The aim of this study was to develop a population pharmacokinetic model and to establish limited sampling strategies (LSSs) enabling accurate estimation of exposure to this drug.

**Methods:**

The study included 15 pediatric patients with malignant and non-malignant diseases, undergoing conditioning regimens prior to HSCT including TREO administered as a 1 h or 2 h infusion at daily doses of 10, 12, or 14 g/m^2^. A population pharmacokinetic model was developed by means of non-linear mixed-effect modeling approach in Monolix® software. Multivariate regression analysis and Bayesian method were used to develop 2- and 3-point strategies for estimation of exposure to TREO.

**Results:**

Pharmacokinetics of TREO was best described with a two-compartmental linear model with proportional residual error. Following sampling schedules allowed accurate estimation of exposure to TREO: 1 h and 6 h or 1 h, 2 h, and 6 h for a TREO dose 12 g/m^2^ in a 1 h infusion, or at 2 h and 6 h or 2 h, 4 h, and 8 h for a TREO dose of 12 g/m^2^ and 14 g/m^2^ in a 2 h infusion.

**Conclusions:**

A two-compartmental population pharmacokinetic model of TREO was developed and successfully used to establish 2- and 3-point LSSs for accurate and precise estimation of TREO AUC_0→∞_.

## Introduction

Allogeneic hematopoietic stem cell transplantation (allo-HSCT) is a procedure aimed at reconstituting normal hematopoiesis in malignancies and non-malignant hematopoietic disorders [1]. A conditioning regimen prior to HSCT is required. It should demonstrate myeloablative and immunosuppressive and, in case of malignant disorders, anti-malignancy properties to prevent graft rejection, graft versus host disease and post-transplant relapse of malignancy. Commonly, the myeloablative conditioning procedure consists of fractionated total body irradiation or myeloablative dose of busulfan combined with high-dose cytostatics, such as etoposide, fludarabine, melphalan, thiotepa or cyclophosphamide [2]. Recent clinical studies indicate that also high-dose treosulfan (TREO), which is a structural analogue of busulfan, demonstrates significant myeloablative and immunosuppressive properties as well as anti-malignant activity in case of hematological malignancies and some solid tumors [3–5]. According to these studies, TREO has relatively mild toxicity profile [5, 6].

**Fig. 1:**
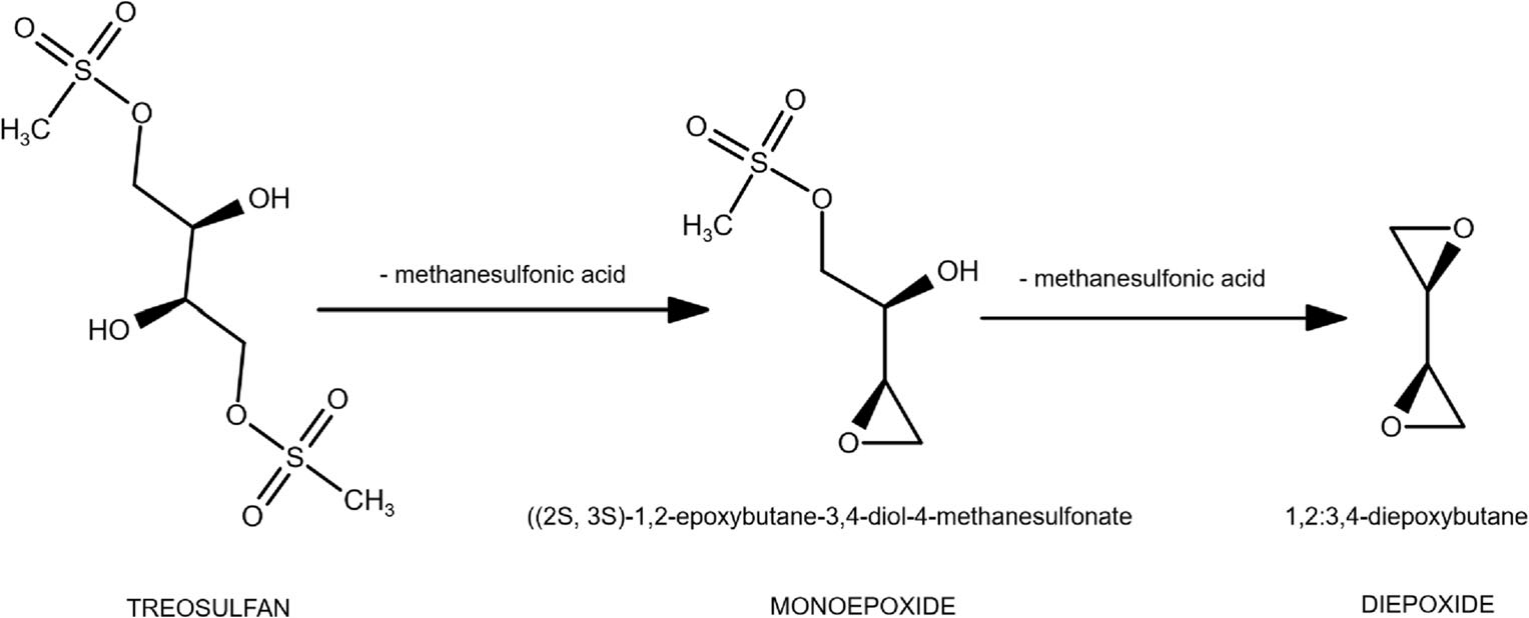
Metabolic activation of treosulfan to its active mono- and diepoxide

TREO is a prodrug and undergoes a non-enzymatic pH-dependent reaction to the monoepoxy- and diepoxytransformers (Fig. 1) [7]. The products of this reaction alkylate DNA at the N7 position of guanine [8]. The optimal dosing regimen of high-dose TREO is still not established and may vary between medical centers [5]. Most commonly, TREO is administered on three subsequent days prior to the transplant procedure (total dose of TREO 30–42 g/m^2^) [4, 6]. A daily dose of TREO (10–14 g/m^2^) is administered in a single infusion.

Since the formation of epoxybutane derivatives from TREO is a non-enzymatic process, it might be assumed that measurement of the prodrug is adequate to describe the alkylating activity [9]. Until now only one limited sampling strategy (LSS) for estimating the exposure to TREO was proposed [10]. However the underlying population pharmacokinetic model assumed a one-compartment linear pharmacokinetics, while other authors suggest that two-compartmental model might more accurately describe changes of TREO concentration over time [9, 11–13]. Therefore the aim of this study was to develop a population pharmacokinetic model and to establish LSSs enabling accurate estimation of exposure to TREO.

## Materials and methods

### Patients’ characteristics

The study included 15 pediatric patients, recruited in years 2007–2011 from the Department of Oncology, Hematology and Pediatric Transplantation at the Poznan University of Medical Sciences and the Department of Pediatric Hematology, Oncology and Bone Marrow Transplantation at the Wroclaw Medical University, with malignant and non-malignant diseases. Detailed patients’ characteristics are presented in Table 1. Conditioning regimens prior to HSCT included TREO administered as a 1 h or 2 h infusion at daily doses of 10, 12, or 14 g/m^2^. The body surface area was calculated at clinical sites by means of Mosteller method [14]. The study protocol was approved by the local Ethical Committee at the Poznan University of Medical Sciences and is in accordance with the 1964 Declaration of Helsinki and its later amendments. Informed consent was obtained from the parents prior to initiating the study.

**Table 1:** Patients’ characteristics. Continuous data are presented as means with standard deviations and minimum-maximum ranges in brackets. Categorical data are presented as counts

Characteristic	Value
Age [years]	7.8 ± 4.9 (0.4–15)
Bodyweight [kg]	26.9 ± 15.7 (7.7–52)
Body surface area [m^2^]	0.95 ± 0.44 (0.25–1.63)
Boys/girls [*n*]	12 / 3
Total daily treosulfan dose and infusion length (*n*)	
10 g/m^2^–1 h	1
12 g/m^2^–1 h	4
12 g/m^2^–2 h	4
14 g/m^2^–2 h	6
Creatinine clearance [ml/min] (*n* = 8)	123 ± 60 (71–239)
Diagnosis	
Hematological malignancies	
ALL	4
AML	1
CML	1
Solid tumors	
NBL	2
ES	2
Non-malignant disorders	
X-ALD	2
DBA	1
SCN	1
WAS	1

*X-ALD* adrenoleukodystrophy, *ALL* acute lymphoblastic leukemia, *AML* acute myeloid leukemia, *CML* chronic myeloid leukemia, *DBA* Diamond-Blackfan anemia, *ES* Ewing’s sarcoma, *NBL* neuroblastoma, *SCN* severe congenital neutropenia, *WAS* Wiskott-Aldrich syndrome

### Sampling protocol and determination of TREO

The samples were drawn in the first day of the therapy from all of the patients. Two different sampling protocols were applied. From 7 patients the full blood samples were drawn at 0.5, 1, 3, 4, 6 and 8 h after the beginning of infusion, while from the remaining 8 patients a more dense sampling was allowed, at 0.5, 1, 1.5, 2, 2.5, 3, 4, 5, 8 and 12 h after the start of infusion. Immediately after collection 50 μl of 1 M citric acid per 1 ml of full blood was added, to avoid ex vivo transformation of TREO to its epoxides. Subsequently, the samples were centrifuged and the obtained plasma was stored at − 20 °C until the analysis.

Concentrations of TREO were determined by a validated HPLC-MS/MS method. The method validation, as well as preliminary pharmacokinetic analysis was published in details elsewhere [12, 15]. Briefly, the applied method allowed accurate determination of TREO in the plasma samples in ranges 0.2–5720 μM (56.6 ng/ml–1.59 mg/ml). The lower limit of quantitation was 0.2 μM. The inter-day and intra-day precision and accuracy were calculated according to regulations of the European Medicines Agency for bioanalytical method validation. The precision of the method, described by the coefficient of variation was 1.8–11.5%, while method accuracy described with the relative error was 0.02–11.8%.

### Development of population pharmacokinetic model

#### Methods and software

The model development was performed in Monolix 2016R1 software (Lixoft SAS, Antony, France, http://lixoft.com/products/monolix/) by means of stochastic approximation of the standard expectation maximization (SAEM) algorithm for non-linear mixed-effects models without approximation. The maximum number of iterations at each stage of population parameter estimation was automatically determined by the algorithm (K_1_ = ‘auto’, K_2_ = ‘auto’). The quality of SAEM algorithm convergence was inspected at each model estimation step. Minimum 4 Markov chains were set at estimation. Conditional means and standard deviations of individual pharmacokinetic parameters were estimated with a Markov Chain Monte Carlo method (MCMC). Improvement in the model fit was evaluated with the likelihood ratio test. The difference in the minimum objective function value (MOFV) of 10.8 (*p* < 0.001) between nested models was considered significant. Also, Akaike information criterion (AIC) and Bayesian Information criterion (BIC) were calculated and models with lower values of AIC and BIC were considered as better fitted to the observed data. Calculation of MOFV was performed by linearization in the initial stages of decision-making, while an importance sampling method was used for the final model selection. Visual examination of goodness-of-fit was based on the following plots: individual (IPRED) and population predicted (PPRED) concentrations versus observed concentrations, individual fits, population weighted (PWRES) and individual weighted residuals (IWRES) versus time and predicted concentrations, normalized prediction distribution errors (NPDE) versus time and predicted concentrations, histograms and quantile-quantile plots.

#### Structural and error model selection

One-, two- and three-compartmental models for intravenous infusion with first-order elimination were examined. Log-normal distribution of pharmacokinetic parameters was assumed and interindividual variability elements (IIV) were described with an exponential model as follows (Eq. 1):

1 $$ {\theta}_{ij}={\theta}_j\times {e}^{\eta ij} $$

where *θ*_ij_ is a value of j-th pharmacokinetic parameter for i-th individual, *θ*_j_ is the population parameter estimate and η_ij_ is a random variable characterizing IIV.

Covariance between IIV elements was inspected after building a structural model. Diagonal, full and partial covariance matrices were examined and significant values were retained in the model. Standard errors of model parameters were calculated by means of the linearization algorithm; however, the values obtained from the final model were obtained by stochastic approximation, which is more time consuming, but at the same time a more precise method [16]. Also, η-shrinkage was calculated with a following equation (Eq. 2):

2 $$ Shrinkage=1-\frac{V\mathrm{ar}\left(\widehat{\eta}\right)}{{\widehat{\omega}}^2} $$

where $\widehat{\eta}$ is the posterior estimate for individuals based on empirical Bayes estimates and $\widehat{\omega}$ is the estimated standard deviation for the corresponding random effect [16]. According to Savic and Karlsson [17], high shrinkage (above 20–30%) is associated with insufficient informativeness of diagnostics based on empirical Bayes estimates, which include IPRED and IWRES.

Additive, proportional and combined (additive and proportional) error models describing residual unexplained variability (RV) were examined and following equation was applied (Eq. 3):

3 $$ {C}_{obs}={C}_{pred}\times \left(1+{\varepsilon}_1\right)+{\varepsilon}_2 $$

where *C*_obs_ and *C*_pred_ are observed and predicted concentrations of treosulfan, ε_1_ is a variable associated with proportional RV and ε_2_ defines additive portion of RV.

#### Covariate selection

Covariate model was established in a forward inclusion-backward elimination manner. Weight and sex were examined as potentially influential covariates. Creatinine clearance was not considered in the covariate analysis due to lack of the data for 7 patients out of total 15 subjects. Weight, a continuous covariate, was standardized to adult bodyweight (70 kg). Also, allometric scaling factor was included for clearance parameters, as suggested by Anderson and Holford [18]. Wald test implemented in Monolix was used to evaluate the covariates and *p* value < 0.05 was considered significant. After including all the significant covariates, a full model was created. Next, the covariates were eliminated from the model in a step-by-step manner. A covariate was retained if the MOFV increased by more than 6.67 (*p* < 0.01). As a result, the final model was obtained and applied in the further analysis.

#### Model evaluation

Due to low number of patients included in the study, internal methods of model validation were applied. Prediction-corrected visual predictive check (pcVPC) was performed for 1000 simulated observations. Contrary to a standard VPC, pcVPC allows accurate visual presentation of the simulation results without losing power due to data stratification [19]. In this approach observed and simulated dependent variables are normalized basing on a typical population prediction for the median independent variable in each bin [20]. 5th, median and 95th percentiles of the simulated data were plotted and compared to the corresponding percentiles of the observed data. The other recommended validation procedure is bootstrapping. Due to the fact that bootstrapping algorithms are not implemented in Monolix, Wings for NONMEM (WFN, version 742, http://wfn.sourceforge.net/, Nick Holford, University of Auckland, New Zealand) with the *mlxbs* script was applied. 1000 bootstrapped datasets were used to determine medians of each estimated parameter, as well as 5th and 95th confidence intervals (CI). Calculated values were compared with those obtained from the final model.

### Development of a LSS

#### Data simulation

A simulation-based approach was applied to develop LSSs for estimation of exposure to TREO in pediatric patients. Firstly, the distribution of significant covariates included in the final model was visually inspected. Deviations from normal distribution were tested with Shapiro-Wilk’s test. Next, a group of 100 virtual patients, with distribution of significant covariates resembling the original population, was simulated in the Statistica 12 software (Stat Soft Inc., Tulsa, OK, USA) by means of a Monte-Carlo method. Subsequently, the time-concentration profiles were simulated for the virtual patients with the S*imulx* function of m*lxR* package (version 3.2.0, Inria Team for the DDMoRe project, http://simulx.webpopix.org/) using the developed final population model. The simulations were performed in R software (version 3.4.0, Foundation for Statistical Computing, Vienna, Austria). In the original study TREO was administered in different dose levels and as a 1 h or 2 h infusion. Therefore, for each virtual patient the concentrations of TREO were simulated after administration of the drug as following: 12 g/m^2^ in 1-h infusion, 12 g/m^2^ in 2-h infusion and 14 g/m^2^ in 2 h infusion. As a result three groups of patients with different types of TREO administration were created, each with 100 individuals. Following sampling times were inspected: 0.5 h, 1 h, 1.5 h, 2 h, 2.5 h, 3 h, 4 h, 6 h, 8 h and12 h after the beginning of the infusion. The suggested sampling times as well as types of TREO administration were based on the original study protocols. All samples in which a predicted concentration was below the LOQ of the applied HPLC-MS/MS method (0.56 ng/ml) were removed from further analysis. Also, the population pharmacokinetic estimates for each subgroups were calculated and compared with the values obtained from the final model.

#### Development of the regression equations for prediction of exposure to TREO by linear regression fitting

In the first step, areas under time-concentration curves from time 0 to infinity (AUC_0 → ∞_) were calculated by means of a non-compartmental method analysis (NCA). The AUC_0 → ∞_ was calculated with a linear-up log-down interpolation method by means of *PKNCA* package (version 0.8.1, https://cran.r-project.org/package=PKNCA) run through the R software. Next, each of the three simulated patient groups were randomly divided into two approximately equal subgroups. First subgroup was a “learning” one, while the second subgroup, which was labeled “validation,” was used to evaluate the predictive performance of the strategies. Multiple regression equations were calculated for models in which two or three samples were required to predict AUC_0 → ∞_. The calculations were performed in R software by means of *leaps* package (version 3.0, https://cran.r-project.org/package=leaps). The models were stratified upon the adjusted coefficient of determination value (*R* ^2^). Models which assumed drawing during the infusion or sampling at12 h after the beginning of infusions were discarded. Finally, best two and three-point models for each type of TREO administration were chosen for further validation.

#### Bayesian estimation of exposure to TREO

The procedure was performed as described by Alsultan et al. [21]. In the analysis, the simulated datasets described in chapter 2.4.1 were used. Also, the best sampling strategies selected by linear regression fitting were applied. First, the estimates from the final model were fixed at the obtained values. Second, the datasets were created in which only the sampling times from the chosen LSSs were left. Then, the individual pharmacokinetic parameters were calculated for each subject. The exposure to TREO for each individual was calculated as following (Eq. 4):

4 $$ {AUC}_{0\to \infty }=\frac{D}{Cl} $$

where *D* is the amount of TREO administered and Cl is total clearance.

#### Evaluation of the predictive performance of LSSs

The prediction performance of LSSs was evaluated as suggested by Sheiner and Beal [22]. Following parameters were calculated for each strategy: relative prediction error (PE), mean relative prediction error (MPE), mean absolute relative prediction error (MAPE) and root mean squared relative prediction error (RMSE). These parameters were estimated using following equations (Eq. 5–8):

5 $$ PE=\frac{{AUC_{pred}}^{(i)}-{AUC_{obs}}^{(i)}}{{AUC_{obs}}^{(i)}}\times 100 $$

6 $$ MPE=\frac{1}{N}{\sum}_i^N\frac{{AUC_{pred}}^{(i)}-{AUC_{obs}}^{(i)}}{{AUC_{obs}}^{(i)}}\times 100 $$

7 $$ MAPE=\frac{1}{N}{\sum}_i^N\frac{\left|{AUC_{pred}}^{(i)}-{AUC_{obs}}^{(i)}\right|}{{AUC_{obs}}^{(i)}}\times 100 $$

8 $$ RMSE=\sqrt{\frac{1}{N}{\sum}_i^N{\left(\frac{AU{C_{pred}}^{(i)}- AU{C_{obs}}^{(i)}}{AU{C_{obs}}^{(i)}}\right)}^2}\times 100 $$

For each LSS, distribution symmetry and range of relative errors was verified [23].

For the linear regression method, the best strategies were used to predict AUC_0 → ∞_ of patients from the “validation” subgroup. Next, also, the LSS equations were used to predict AUC_0 → ∞_ calculated from the acquired experimental data, and the cumulative predictive performance was calculated for the best 2- and 3-point strategies as presented above. For the Bayesian method, the chosen strategies were used to predict AUC_0 → ∞_ of all patients from a given dosing subgroup (100 patients in each).

## Results

### Population pharmacokinetic analysis

**Fig. 2:**
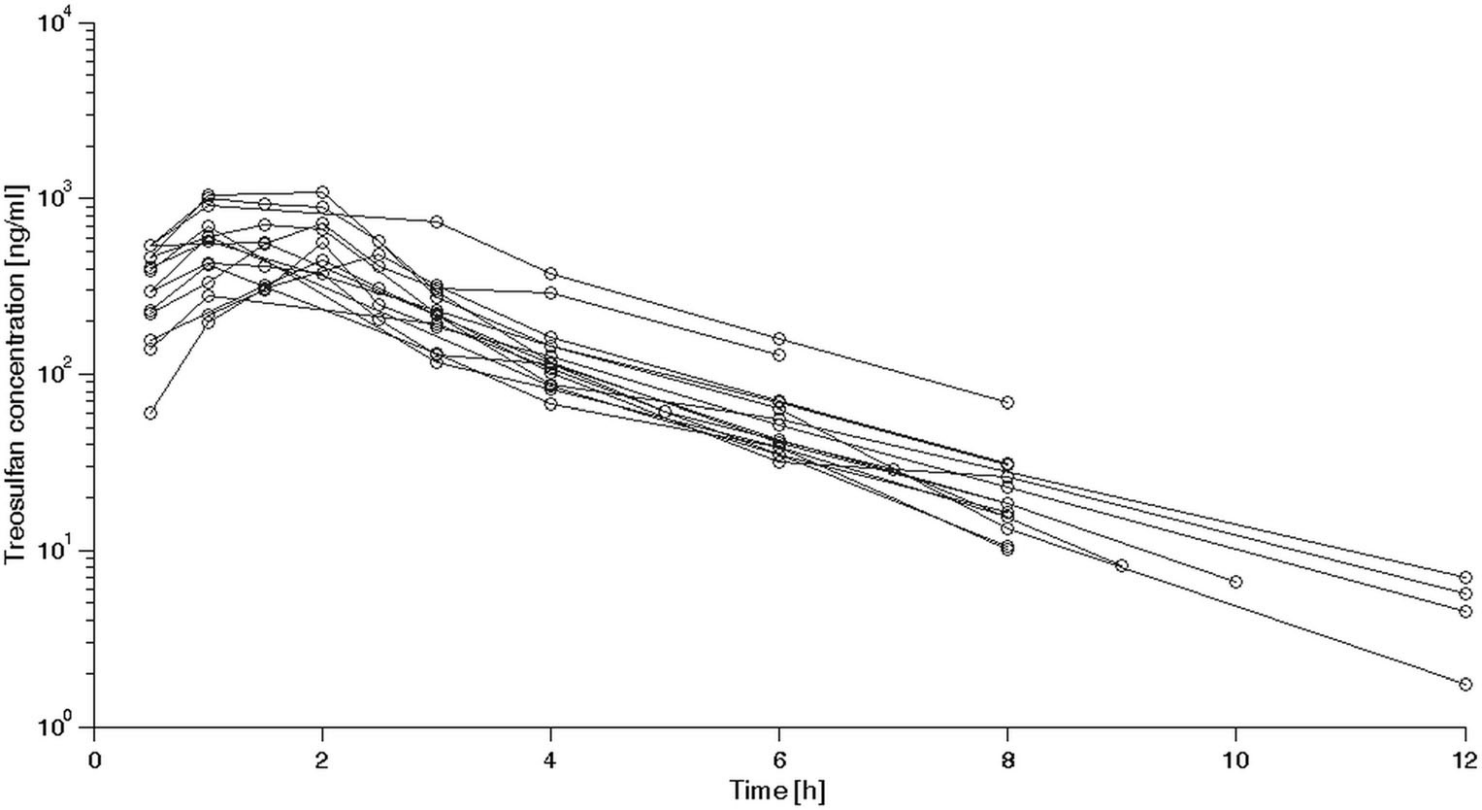
Spaghetti plot of treosulfan concentrations vs. time acquired for patients included in the study

A total of 110 samples were obtained from the patients and analyzed in the study. All concentrations of TREO were above the LOQ of the HPLC-MS/MS method. A spaghetti plot with individual time-concentration curves is presented in Fig. 2. It was found that the experimental data were best described by a linear two-compartmental model with a proportional error (63.76 decrease of MOFV), where V_1_ and V_2_ describe central and peripheral compartment volumes, respectively, Cl is clearance and *Q* is an intercompartmental clearance. Analysis of covariance matrix showed that there is a significant covariance between random variability of Cl and V_1_. The only covariate included in the final model was patient’s weight, as described by following equation (Eq. 9):

9 $$ {\theta}_{ij}={\theta}_j\times {\left(\frac{BW_i}{70}\right)}^{\beta}\times {e}^{\eta ij} $$

where θ_ij_ is a value of j-th pharmacokinetic parameter for i-th individual, θ_j_ is the population parameter estimate, BW_i_ is a bodyweight of the i-th individual centered on a typical weight of 70 kg, while β is a scaling exponent and η_ij_ is a random variable characterizing IIV. The values of the exponents were first estimated and equaled 0.804 for Cl, 0.959 for V_1_ and 0.925 for V_2_. For subsequent analysis, these values were fixed to most widely used values of 1 for volume parameters or to 0.75 for clearance. Also, the IIV parameters were evaluated for CL, V_1_ and Q only. In the modeling process, the standard error for the estimated V_2_ IIV was over 100%. Hence it was decided to remove the IIV on V_2_ from the model. It was found that addition of bodyweight as a covariate decreased the IIV of CL (65.3 to 25.5%) and V_1_ (84.5 to 51.4%). Interestingly, addition of bodyweight as a covariate for Q increased the MOFV and worsened the model fit. Therefore this relationship was not included in the model. The estimates derived from the final pharmacokinetic model are presented in Table 2. Calculated η-shrinkage was 1% for Cl, 19% for V_1_ and 18% for Q.

**Table 2:** Final estimates of pharmacokinetic parameters

Parameter	Final model estimate (%RSE)	Bootstrapped estimate (95% CI)
Typical value		
Cl [l/h/70 kg]	14.7 (6.9)	14.78 (14.70–14.84)
β_Cl, weight_	0.75 (fixed)	0.75 (fixed)
V_1_ [l/70 kg]	26.0 (14.0)	25.94 (25.70–26.18)
β_V1, weight_	1 (fixed)	1 (fixed)
Q [l/h]	2.25 (22.2)	2.63 (2.53–2.72)
V_2_ [l/70 kg]	9.93 (9.0)	9.89 (9.74–10.05)
β_V2, weight_	1 (fixed)	1 (fixed)
IIV [%]		
ωCl	25.5 (19.8)	24.0 (23.6–24.3)
ωV_1_	51.4 (20.0)	50.8 (50.2–51.3)
ωQ	38.6 (52.3)	32.7 (31.6–33.7)
ωCl-V_1_	71.4 (20.7)	68.9 (67.7–70.1)
Residual proportional error	0.188 (9.01)	0.184 (0.182–0.185)

*RSE* relative standard error, *CI* confidence interval, *Cl* clearance, *V*_*1*_ central compartment volume, *Q* intercompartmental clearance, *V*_*2*_ peripheral compartment volume, *IIV* interindividual variability

**Fig. 3:**
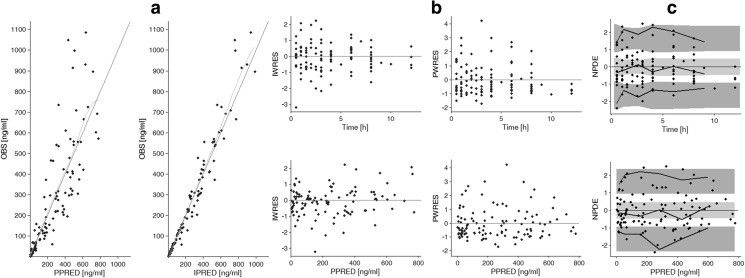
Goodness-of-fit plots for the final pharmacokinetic model. Panel A illustrates observed (OBS) vs. population predicted (PPRED) treosulfan concentrations and OBS vs. individual predicted (IPRED) treosulfan concentrations with an identity line and smooth. Panel B presents individual-weighted residuals (IWRES) vs. time and PPRED, population-weighted residuals (PWRES) vs. time and PPRED. Panel C presents normalized prediction distribution errors (NPDE) vs. time and PPRED with bold lines as 5th, median and 95th percentile of observed concentrations, light gray area as 50% interval of simulated data and dark gray areas as 95% intervals of simulated data

Figure 3 presents basic goodness-of-fit diagnostic plots of the final model. Observed concentrations (OBS) plotted vs. population predicted (PPRED) and individual predicted concentrations (IPRED) are scattered randomly around the line of identity and the spline is close to the identity line (Fig. 3A). Plots of IWRES and PWRES vs. time and PPRED are presented in Fig. 3B. For both types of residuals, the points are randomly scattered along the y = 0 line and no significant trends are visible. Moreover, most points fall within ±2 standard deviation with only a few points with deviations larger than 3. Simulation-based plots of NPDE vs. time and PPRED (Fig. 3C) show that the 5th, median and 95th percentiles of empirical data (solid lines) fall within the corresponding percentiles of the predictions (light and dark gray areas).

**Fig. 4:**
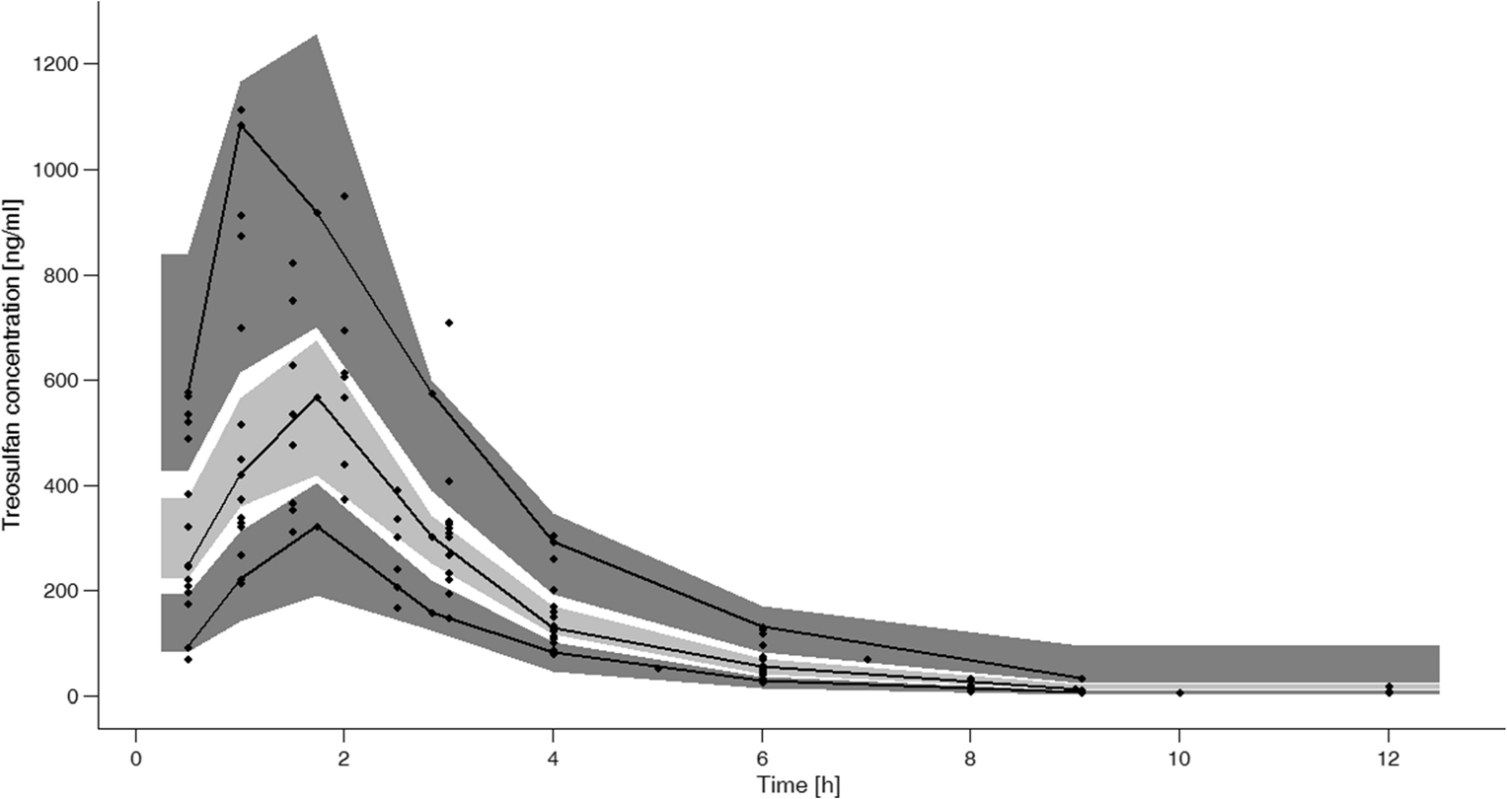
Prediction-corrected visual predictive check (pcVPC) with dots as observed treosulfan concentrations, bold lines as 5th, median and 95th percentile of observed concentrations, light gray area as 50% interval of simulated data and dark gray areas as 95% intervals of simulated data

Performed pcVPC is presented in Fig. 4. The solid black lines, which represent 5th, median and 95th percentile of observed data fall within the areas representing respective prediction intervals of the simulated data. There are only 2 points which fall outside the plotted prediction intervals.

The results of bootstrapping are presented in Table 2. Calculated means as well as the 5th–95th confidence intervals are very similar to the estimates obtained with the SAEM algorithm for the primary dataset. The largest discrepancy was noted for the IIV of Q. However, the calculated standard error for this particular parameter was large (52.3%) and also small size of study group might account for the difference in the results.

### Performance of selected LSSs

Covariate analysis showed that the patient’s bodyweight is an important cofactor. Visual inspection of the data and results of Shapiro-Wilk’s test (*p* = 0.182) it was assumed that the bodyweight was normally distributed. 100 patients with mean body weight 26.26 ± 16.54 kg (min. 5.8 kg – max. 53.90 kg) were simulated with a Monte-Carlo method. Next, body surface area was estimated with a following equation (Eq. 10) [24]:

10 $$ BSA=4,688\kern0.5em {BW}^{\left(0,8168-0,0154\kern0.5em \log BW\right)} $$

where BSA is body surface area in cm^2^ and BW is bodyweight in g. Obtained body surface areas were used to calculate the exact dose of TREO (12 g/m^2^ or 14 g/m^2^) for the simulation analysis.

For all three types of TREO administration the population pharmacokinetic estimates were not significantly different from the values obtained from the final model.

The best strategies and their performance for estimating exposure to TREO, based on the simulation study, are presented in Table 3. For 1-h infusion of 12 g/m^2^ of TREO, the best strategies assumed sampling at 1 h and 6 h or at 1.5 h, 2 h and 6 h after the beginning of the infusion. For 2-h infusion of 12 g/m^2^ an accurate prediction of AUC_0 → ∞_ required determination of TREO concentration in samples drawn 2 h and 6 h or 2 h, 3 h and 8 h after the beginning of infusion. While for 2-h infusion of 14 g/m^2^ of the drug, sampling at 2 h and 6 h, or at 2 h, 4 h and 8 h was needed for the most accurate estimation of exposure. The *R* ^2^ is very close to the unity value and the prediction errors are overall very small. Noteworthy, the variability observed in the Bayesian prediction method is higher, as well as the prediction errors. Also, approximately 2% of the predictions based on this method were outside the 20% error boundary.

**Table 3:** Performance of chosen two and three-point LLSs based on linear regression fitting and Bayesian estimation for prediction of exposure to treosulfan after different dosing regimens

Equation (if applicable)	*R* ^2^	PE > 20%	PE < 20%	MPE [%]	MAPE [%]	RMSE [%]
12 g/m^2^ in 1 h infusion						
AUC_pred_ = 0.86×C_1 h_ + 1.93×C_2 h_ + 7.47×C_6 h_ – 42.2	0.9978	0	0	0.13	1.13	1.36
AUC_pred_ = 2.78×C_1.5 h_ + 6.62×C_6 h_ – 45.6	0.9743	0	0	− 0.12	2.78	4.09
Bayesian estimation from C_1 h,_ C_2 h,_ C_6 h_	–	0	0	− 0.95	3.55	4.39
Bayesian estimation from C_1.5 h,_ C_6 h_	–	0	0	− 0.36	6.58	7.86
12 g/m^2^ in 2 h infusion						
AUC_pred_ = 1.82×C_2 h_ + 1.51×C_3 h_ + 9.39×C_8 h_ – 24.1	0.9993	0	0	0.11	0.78	0.93
AUC_pred_ = 2.10×C_2 h_ + 7.73×C_6 h_ + 2.94	0.9929	0	0	− 0.29	1.40	1.67
Bayesian estimation from C_2 h,_ C_3 h,_ C_8 h_	–	0	0	− 0.61	4.35	5.60
Bayesian estimation from C_2 h,_ C_6 h_	–	2	0	0.50	5.37	7.56
14 g/m^2^ in 2 h infusion						
AUC_pred_ = 2.01×C_2 h_ + 2.15×C_4 h_ + 7.68×C_8 h_ – 9.07	0.9998	0	0	− 0.29	0.45	0.62
AUC_pred_ = 2.07×C_2 h_ + 7.57×C_6 h_ + 42.6	0.9972	0	0	− 0.29	1.37	1.61
Bayesian estimation from C_2 h,_ C_4 h,_ C_8 h_	–	1	0	− 0.92	5.13	6.68
Bayesian estimation from C_2 h,_ C_6 h_	–	1	0	0.24	5.97	8.12

*LSS* limited sampling strategy, *R* ^*2*^ adjusted coefficient of determination, *PE* relative prediction error, *MPE* mean relative prediction error, *MAPE* mean absolute relative prediction error, *RMSE* root mean squared relative prediction error, *AUC*_*pred*_ predicted area under time-concentration curve, *C*_*nh*_ concentration of treosulfan measured n hours after the beginning of infusion

The performance of the proposed LSSs was also evaluated by comparison of observed and predicted AUC_0 → ∞_ in the experimental group (Table 4). Bias in prediction of this parameter was within ±15% for all patients. Only 3-point strategies in which the exposure was estimated with Bayesian methods had RMSE slightly above 15%. Unfortunately, due to heterogeneity in the sampling designs and lack of samples in some time points, prediction of AUC_0 → ∞_ was not possible for all of the patients, while for some individuals prediction was calculated only by means of 2-point strategies.

**Table 4:** Performance of proposed LSSs for prediction of AUC_0 → ∞_ in the primary group of patients

	Linear regression method		Bayesian method	
	2-point strategies (*n* = 7)	3-point strategies (*n* = 5)	2-point strategies (*n* = 7)	3-point strategies (*n* = 5)
PE > 20%	0	0	0	0
PE < − 20%	0	0	0	0
MPE [%]	0.89	− 6.25	1.04	− 2.05
MAPE [%]	8.43	9.72	11.07	13.51
RMSE [%]	9.33	11.34	12.56	15.83

*LSS* limited sampling strategy, *PE* relative prediction error, *MPE* mean relative prediction error, *MAPE* mean absolute relative prediction error, *RMSE* root mean squared relative prediction error

## Discussion

There is an increasing interest in use of TREO as a basic agent in conditioning regimens prior to hematopoietic stem cell transplantation, both in pediatric and adult populations [3, 25, 26]. The aim of this study was to determine a population pharmacokinetic model for TREO basing on the data acquired for pediatric population and to develop LSSs for estimation of AUC of this drug.

According to Scheulen et al. [27], the pharmacokinetics of TREO is linear in a wide range of doses (20–56 g/m^2^) therefore it was possible to pool patients with different dose levels and it might be assumed that the dose would not have an impact on the estimations. It was found that the pharmacokinetics of TREO was best described with a linear two-compartmental model with a proportional residual error. The covariate analysis showed that bodyweight was significantly associated with the estimated values of Cl, V_1_ and V_2_. Interestingly, visual inspection of data and analysis of MOFV did not support inclusion of bodyweight as an important covariate of Q. Although the explanation of this phenomenon remains unknown, one of the reasons might be unique metabolic activation of TREO. Scaling of parameters, especially allometric scaling of clearance parameters, describes an increase in the metabolic rate [28]. At the same time activation of TREO is a pH- and temperature-dependent process and does not require enzymatic conversion. Therefore the lack of relationship between Q and bodyweight might, to some extent, reflect this pathway. The conversion of TREO to epoxides might occur throughout the whole body both in the central and peripheral compartments. However, more studies are needed, especially the combined parent-metabolite modeling approach, to sufficiently explore this observation. The other tested covariate, patient’s sex, was not found significant. One explanation of these observations might be the fact that the study group included three girls only and this number might be insufficient to observe potentially existing relationships between estimated parameters. Also, some authors note that patient’s sex might be an important factor in children older than 12 years of age [29]. In the study group only four children (three boys and one girl) could be qualified into this category and therefore a more thorough investigation of this potential covariance could not be performed. Previous studies on pharmacokinetics of TREO indicate that up to 39% of total dose of this drug is excreted renally [12]. As a consequence, parameters which describe renal function such as creatinine clearance might be important covariates for Cl. However, in the present study, this factor was not included in the analysis. First of all, data on creatinine clearance was available for eight children (53% of total). Several methods to overcome the problem of missing data were considered in this analysis—exclusion of patients with lack of information on creatinine clearance or imputation with a median value [30]. Although removing individuals with missing covariate data gives relatively unbiased results, the estimates are less precise. Imputation of a median value might significantly bias the results of the study. Noteworthy, no significant trends were observed in the plots of neither Cl vs. creatinine clearance nor IIV_Cl_ vs. creatinine clearance. Also, the available data on creatinine clearance indicate that the renal function in patients could be described as normal. Therefore, to investigate the influence of this parameter on pharmacokinetics of TREO a larger study group would be necessary.

The parameter estimations obtained in the present study are similar to the results reported by other authors. In adult populations [9, 13, 26] estimated total clearance ranged from 8.7 l/h to 13.5 l/h, while mean steady-state volume of distribution (V_ss_), which represents both central and peripheral compartments, was ranging from 19.5 l to 34 l. The obtained values are also similar to the ones presented by ten Brink et al. (V_c_/70 kg = 43.05 l, Cl/70 kg = 16.12 l/h) [10]; however, it has to be noted that the model assumed by those authors is one-compartmental. The IIV of calculated parameters was relatively large, reaching up to 51.4%. According to the presented goodness-of-fit plots (Fig. 3), pcVPC (Fig. 4) and bootstrap analysis (Table 2), proposed model adequately describes changes of TREO concentration over time.

Overall, the results are in some contrast with the results of ten Brink et al. [10] who developed a one-compartment model. These authors note that using a two-compartmental model did not sufficiently lower the MOFV (< 10.8 decrease in the MOFV). However, a systematic bias is noticeable in the conditional-weighted residuals vs. time graphs presented by these authors. It indicates that the addition of peripheral compartment might had improved the model. Therefore, the model developed in the present study might be superior to the previous one and allows better prediction of changes in TREO concentration over time.

The best LSSs developed on a basis of the proposed population pharmacokinetic model were shown to have good predictive properties (Table 3). Interestingly, the equations proposed for estimation of AUC_0 → ∞_ on a 2-sample basis were very similar for 2-h infusions of 12 g/m^2^ and 14 g/m^2^. This is an understandable consequence of the assumed linearity of TREO pharmacokinetics. Moreover, the strategies were applied to predict the AUC_0 → ∞_ in the experimental group and the predictive performance was acceptable.

As administration of TREO in HSCT in pediatric populations is still a relatively new issue, because standard procedures rather include busulfan, a uniform system for estimation of exposure is yet to be evaluated. Also, the exact and detailed protocols for monitoring of TREO have also not been published yet, beside the work by ten Brink et al. [10]. TREO itself is a prodrug, and its pharmacological effect is dependent on the epoxytransformers. Since the concentrations of TREO are much higher than the concentrations of its “metabolites,” they are much easier to monitor. Therefore the monitoring procedure based on the prodrug concentration was proposed. To our knowledge, no studies were published which would bind directly the efficacy and safety of TREO with its pharmacokinetics and several authors point out the need of further studies in this particular area [4, 5]. It might be possible that the levels of TREO epoxides have higher predictive value for safety, toxicity and efficacy of this treatment. However, exact data which combine follow-up data and the epoxides concentrations would be required for this assessment.

The major limitation of the study is small sample size. Therefore, an external validation procedure is required prior to employing the proposed LSS in the clinical practice.

## Conclusions

In conclusion, in the present study a two-compartmental population pharmacokinetic model of TREO was developed and successfully used to establish 2- and 3-point LSSs for accurate and precise estimation of TREO AUC_0 → ∞_.
